# Low-Frequency vs. Theta Burst Transcranial Magnetic Stimulation for the Treatment of Chronic Non-fluent Aphasia in Stroke: A Proof-of-Concept Study

**DOI:** 10.3389/fnagi.2021.800377

**Published:** 2022-01-14

**Authors:** Ting-Yu Chou, Jia-Chi Wang, Mu-Yun Lin, Po-Yi Tsai

**Affiliations:** ^1^Department of Physical Medicine and Rehabilitation, Taipei Veterans General Hospital, Taipei, Taiwan; ^2^School of Medicine, National Yang Ming Chiao Tung University, Taipei, Taiwan

**Keywords:** transcranial magnetic stimulation, intermittent theta burst stimulation, stroke, aphasia, neuromodulation

## Abstract

**Background:**

Although low-frequency repetitive transcranial magnetic stimulation (LF-rTMS) has shown promise in the treatment of poststroke aphasia, the efficacy of high-frequency rTMS (HF-rTMS) has yet to be determined.

**Purpose:**

We investigated the efficacy of intermittent theta burst stimulation (iTBS) in ameliorating chronic non-fluent aphasia and compared it with that of LF-rTMS.

**Methods:**

We randomly assigned patients with poststroke non-fluent aphasia to an ipsilesional iTBS (*n* = 29), contralesional 1-Hz rTMS (*n* = 27), or sham (*n* = 29) group. Each group received the rTMS protocol executed in 10 daily sessions over 2 weeks. We evaluated language function before and after the intervention by using the Concise Chinese Aphasia Test (CCAT).

**Results:**

Compared with the sham group, the iTBS group exhibited significant improvements in conversation, description, and expression scores (*P* = 0.0004–0.031), which characterize verbal production, as well as in auditory comprehension, reading comprehension, and matching scores (*P* < 0.01), which characterize language perception. The 1-Hz group exhibited superior improvements in expression, reading comprehension, and imitation writing scores compared with the sham group (*P* < 0.05). The iTBS group had significantly superior results in CCAT total score, matching and auditory comprehension (*P* < 0.05) relative to the 1-Hz group.

**Conclusion:**

Our study findings contribute to a growing body of evidence that ipsilesional iTBS enhances the language recovery of patients with non-fluent aphasia after a chronic stroke. Auditory comprehension was more preferentially enhanced by iTBS compared with the 1-Hz protocol. Our findings highlight the importance of ipsilesional modulation through excitatory rTMS for the recovery of non-fluent aphasia in patients with chronic stroke.

**Clinical Trial Registration:**

[www.ClinicalTrials.gov], identifier [NCT03059225].

## Key Summary Points

- Both the iTBS and LF-rTMS enhances language recovery in chronic poststsroke aphasia.
- The ipsilesional iTBS achieved a superior outcome in overall language performance.
- Auditory comprehension was more enhanced by iTBS compared with the 1-Hz protocol.
- Our findings highlight the importance of excitatory rTMS protocol in chronic aphasia.

## Introduction

Stroke is a leading cause of disability and the third most frequent cause of death globally (Wade et al., 1986; Dionisio et al., 2018). Among the most debilitating consequences of stroke, aphasia can have a severe impact on health outcomes and quality of life. Language recovery depends mainly on the severity of the condition and localization in the dominant hemisphere (Wade et al., 1986; Naeser and Palumbo, 1994; Hilari et al., 2012). Speech and language therapy, the gold-standard treatment for aphasia, requires active participation and repetition; however, it is usually associated with unsatisfactory recovery (Brady et al., 2016). Novel rehabilitation approaches such as neuromodulation with non-invasive brain stimulation, particularly repetitive transcranial magnetic stimulation (rTMS), have opened a new era in neurorehabilitation (Hilari et al., 2012).

In chronic non-fluent aphasia after stroke, studies have frequently described compensatory activations of the right homolog of the language-related area in the brain during language tasks, and this overactivation may not represent entirely beneficial neuroplasticity (Benson, 1986; Thiel et al., 2006). Alternatively, according to interhemispheric imbalance modeling, this overactivity may play a deleterious role for optimal language recovery owing to the loss of normal transcallosal inhibition from the damaged center (Kapoor, 2017; Sebastianelli et al., 2017; Harvey et al., 2019). A painless non-invasive technology, rTMS has drawn considerable attention in efforts aimed at ameliorating motor dysfunction, aphasia, dysphagia, and visuospatial neglect in patients with stroke (Ward, 2005; Feldman, 2009; Naeser et al., 2010a; Tsai et al., 2014, 2020; Saxena and Hillis, 2017). Low-frequency rTMS (LF-rTMS, ≤ 1 Hz) is commonly used to reduce cortical excitability (Barwood et al., 2011); by contrast, high-frequency rTMS (HF-rTMS, ≥ 5 Hz) is applied to increase cortical excitability by promoting synaptic transmission. According to the theory of long-term potentiation, long-term depression, and paradoxical functional facilitation (Kapur, 1996), rTMS harnesses neuroplasticity and ameliorates interhemispheric imbalance, leading to effective language recovery (Wu et al., 2000; Heiss and Thiel, 2006; Calautti et al., 2007; Cramer, 2008; Griffis et al., 2016; Dionisio et al., 2018).

Currently, LF-rTMS is mainstream and the most commonly used protocol for the treatment of poststroke aphasia (Barwood et al., 2011; Ren et al., 2014; Li et al., 2020; Yao et al., 2020). According to International Federation of Clinical Neurophysiology guidelines on the therapeutic use of rTMS, evidence B (“probably”) was proposed for LF-rTMS conducted on the right pars triangularis in patients with chronic non-fluent aphasia (Lefaucheur et al., 2014). This LF-rTMS protocol leads to substantial language improvement, including naming and expressive abilities (Hu et al., 2018; Harvey et al., 2019). However, a meta-analysis indicated that LF-rTMS yielded no significant effect on repetition and auditory comprehension (Li et al., 2020). In our experience, limitations exist for contralesional modulation in its efficacy in improving auditory or verbal comprehension outcomes in patients with non-fluent aphasia (Hu et al., 2018).

In contrast to the LF-rTMS protocol, which has undergone large-scale research, the efficacy of HF-rTMS has yet to be fully studied. For optimal language recovery, reorganization in the affected hemisphere is a crucial step in achieving long-term outcomes (Karbe et al., 1998; Heiss and Thiel, 2006; Thiel et al., 2006; Griffis et al., 2016). The direct stimulation of residual language nodes or unmasking of potential perilesional nodes may be more effective than homotopic compensatory recruitment. The mechanism underlying the effects of HF-rTMS on neuroplasticity could be associated with modulations of synaptic transmission, neurotransmitter production, growth factor generation, and gene expression, which may lead to perilesional reorganization (Bates and Rodger, 2015). Studies administering excitatory rTMS to patients with chronic aphasia have reported notable improvements in language abilities (Szaflarski et al., 2011, 2018). A case study demonstrated that 10-Hz rTMS treatment over the left inferior frontal gyrus (IFG) engendered both short- and long-term improvements in repetition, naming, and comprehension tests (Dammekens et al., 2014). Intermittent theta burst stimulation (iTBS) applied in 8 patients with chronic aphasia resulted in clinical improvements in verbal fluency along with increased left hemispheric recruitment as observed in functional magnetic resonance imaging (fMRI) (Griffis et al., 2016). Previous studies have not compared ipsilesional HF-rTMS with contralesional LF-rTMS in terms of language enhancement. In functional restoration, a head-to-head comparison with a randomized controlled trial (RCT) design is warranted to advance our understanding of the differential effects of HF-rTMS vs. LF-rTMS.

On the basis of clinical, neurobiological, and neuroimaging evidence that LF- and HF-rTMS exhibit positive effects on language recovery, we hypothesized that the effect of ipsilesional iTBS would differ from that of contralesional LF-rTMS in the treatment of poststroke aphasia. Accordingly, the aim of this study was to apply a RCT design to comprehensively compare the effects of these 2 protocols in ameliorating non-fluent aphasia in patients with chronic stroke.

## Materials and Methods

### Participants

**FIGURE 1:**
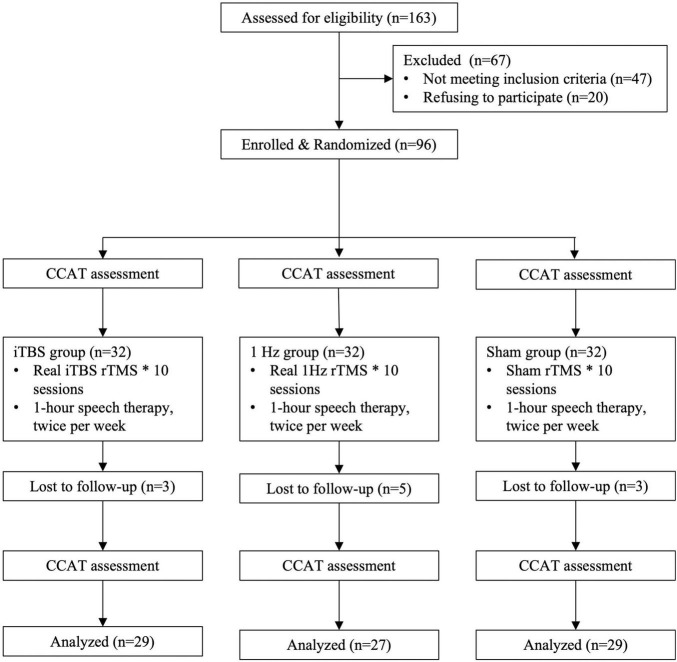
Flowchart for recruitment, group allocation, treatment allocation, follow-up, and analysis.

To select participants for inclusion in this study, we consecutively screened 157 stroke patients with aphasia who visited the rehabilitation clinic of a tertiary medical center or were admitted to its stroke ward. Of the screened patients, 47 did not meet the inclusion criteria and 20 declined to participate. Therefore, the remaining 90 patients who met the inclusion criteria were randomized to 3 groups initially (Figure 1).

The inclusion criteria were as follows: (a) receiving a diagnosis of aphasia secondary to a first-ever left hemispheric ischemic or hemorrhagic stroke, confirmed by either computed tomography or MRI; (b) having a stroke at least 3 months previously and being in a stable medical and cognitive condition; (c) having no seizure history; (d) having no intracranial occupying lesion, such as a brain tumor, according to imaging results; (e) having no confirmed neurodegenerative diseases; (f) having no visual field deficit or emotional problems; and (g) having no TMS contraindication (metallic intracranial device, pacemaker, or other electronic device implantation). The rTMS protocols were in accordance with the safety guidelines for rTMS applications (Rossini et al., 1994). Aphasia type was defined by a physician or speech therapist on the basis of the Concise Chinese Aphasia Test (CCAT). This study was performed in accordance with the Declaration of Helsinki and was approved by the Institutional Review Board of Taipei Veterans General Hospital (IRB No., 201405003A); all patients provided written informed consent before participation.

### Design

We conducted a randomized, single-blind, sham-controlled study with blinded outcome assessment. The randomization order was computer generated and concealed in sequentially numbered opaque envelopes by an independent statistician. The 90 patients were randomly assigned to an iTBS (*n* = 30), 1-Hz (*n* = 30), or sham (*n* = 30) group (Figure 1).

Sham or actual stimulation was applied in daily sessions over 10 consecutive weekdays. All participants continued their conventional speech rehabilitation program and other medical treatments regardless of group assignment. Moreover, all participants undertook the same amount of 1-h speech therapy conducted twice a week by a therapist blinded to group allocation. The speech therapy program was based on constraint-induced therapy, a type of use-dependent treatment approach (Pulvermüller et al., 2001; Kristensen et al., 2015). The content included expressive production, semantic training, phonemic training, repetition, naming, conversation, picture description, and phrase generation tasks under the principles of both forcing patients to use verbal language and intensive practice. The time frame of speech therapy was not related to the rTMS schedule. The training difficulty level was adjusted according to individual communicative capacity with the aim of reaching adequate training intensity, which was evaluated weekly. Other methods, such as drawing, gesturing, or melody intonation, were not encouraged during the training course.

### Determining Resting Motor Threshold

We applied rTMS by using a Rapid2 (Magstim) device and a 70-mm figure-8 coil. We recorded the motor-evoked potentials (MEPs) bilaterally from the first dorsal interosseous (FDI) hand muscles by using surface Ag/AgCl electrodes. A Keypoint electromyograph machine (Dantec) was connected to the TMS stimulator to record the MEP signals. The amplified (100–1 mV/div) and bandpass-filtered (20–2,000 Hz) signals were digitized at a sampling rate of 20 kHz.

The participants were asked to sit in a chair upright and relaxed with eyes open. The coil was displaced over the motor cortex covered by a grid of 49 positions until the largest consistent MEP response from the contralateral FDI was recorded. The resting motor threshold for the FDI, as the minimal intensity, was that at which an MEP of at least 50 mV was elicited in 5 of 10 consecutive sessions (Rossini et al., 1994).

### Stimulation Protocols

Each patient received 10 days of rTMS treatment, administered on workdays for 2 consecutive weeks. Intermittent TBS treatment consisted of 3 pulses of 50-Hz bursts repeated at 5 Hz (2 s on and 8 s off) for a total of 190 s (Huang et al., 2005). We applied 1-Hz rTMS trains for 20 min. A placebo coil (Magstim) with less than 5% magnetic output with an audible click on discharge was applied as the sham stimulation. None of the participants had previously experienced rTMS; hence, they were thoroughly blinded with regard to receiving treatment or sham stimulation. The intensity of the 1-Hz rTMS was set at 90% of the resting motor threshold (rMT), and that of the iTBS was set at 80% of the rMT, which matched the participants’ maximum tolerance.

We used a frameless stereotaxic system (Brainsight, Rogue Research, Montreal, Canada) to guide the localization of the following target areas: the bilateral posterior pars triangularis (PTr), Brodmann area 45 (BA45, which is defined as the area rostral to the vertical ascending ramus and caudal to the triangular sulcus) (Devlin et al., 2003; Naeser et al., 2005). If the diagonal sulcus was present, then PTr posterior was defined as the gyrus rostral to the diagonal sulcus and caudal to the vertical ascending ramus. The sham stimulation was applied over the ipsilesional PTr. The participants received 3-T MRI performed using a General Electric (Milwaukee WI) scanner under the following T1-weighted imaging conditions: TR = 8.20 ms; TE = 3.24 ms; slice number = 180 slices; slice thickness = 4 mm; field of view = 23 × 23 cm^2^; and magic angle turning = 256 × 256. Brain lesion locations and the presence of periventricular and subcortical hyperintensities indicating white matter lesions were registered during the MRI examination.

### Assessment of Language Performance

A speech therapist blinded to group allocation evaluated the language performance of the participants shortly before the first intervention session (baseline) and on the day after the completion of the protocol by using the CCAT (Chung et al., 1998). The CCAT, the only standardized linguistically and culturally neutral assessment for native Mandarin Chinese speakers, tests all language modalities. It comprises the following subtests used to assess verbal output: conversation, description, expression, and repetition tests. Imitation and spontaneous writing are used to test writing output. Perception ability is assessed using the following subtests: auditory, reading comprehension, and matching tests. The scoring of the CCAT subtest ranges from 0 to 12, where 0 indicates the maximum abnormality. CCAT total score is calculated as the sum of subtest scores.

### Statistical Analysis

We used the G*Power program (v3.1.9.2; Franz Faul, University of Kiel, Kiel, Germany) to calculate the minimum sample size. Based on clinical experience, we adopted Cohen *d*-value of 0.4, which indicate a medium effect size. In total, 51 participants were required to achieve a statistical power of 80% with an alpha-error of 0.05. Anticipating 15% dropout and non-compliance, we determined that at least 20 participants were needed in each group.

We compared the baseline assessments and biographic data of the groups by using one-way analysis of variance (ANOVA) for continuous data and χ^2^-tests for categorical data as appropriate. To determine improvements in CCAT total and subtest scores, we used a paired *t*-test for intragroup comparisons and ANOVA for intergroup comparisons with corrected variances for age and sex. The level of significance was set at *P* < 0.05. We conducted these analyses using SPSS version 26.

## Results

### Demographic and Clinical Characteristics

Table 1 lists participant demographic and clinical variables. We observed no differences between the groups in terms of baseline features, such as time poststroke, aphasia type, stroke type, lesion site and CCAT scores (*P* > 0.05; Table 1). Two participants in the TBS group reported dizziness at first, but their discomfort subsided once the stimulation intensity was reduced by 2%. During the treatment sessions, 1 participant in the iTBS group was lost to follow-up, and 3 in the 1 Hz group and 1 in the sham group withdrew for personal reasons, resulting in a total of 85 participants. None of the participants reported adverse effects during the experimental period.

**TABLE 1:** Demographic data and clinical variables of participants.

Characteristics	All	iTBS	1 Hz	Sham	
	(*n* = 85)	(*n* = 29)	(*n* = 27)	(*n* = 29)	*p*-value
Male sex, n (%)	54 (64)	15 (52)	19 (70)	20 (69)	0.265
Age (years), mean (SD)	60.5 (13.6)	62.7 (12.7)	56.9 (13.2)	61.6 (14.7)	0.25
Post-stroke duration (months), mean (SD)	15.8 (22.1)	17.6 (20.8)	13.2 (21)	16.5 (24.6)	0.744
Stroke type (Ischemic/Hemorrhage)	62/23	20/9	22/5	20/9	0.481
Lesion site (Cortical/Subcortical/Mixed)	54/24/7	15/10/4	21/5/1	18/9/2	0.318
Aphasia type (Broca/Transcortical motor/Transcortical mixed/Global)	35/22/11/17	9/10/4/6	13/8/2/4	13/4/5/7	0.489
Pre-rTMS CCAT Total Score, mean (SD)	66.7 (21.1)	67.3 (19.4)	69.5 (20.9)	63.3 (23)	0.53

*CCAT, Concise Chinese Aphasia Test; rTMS, repetitive transcranial magnetic stimulation. (Continuous data: ANOVA; Categorical data: χ^2^-test).*

### Group-Wise Improvement

After 10 rTMS sessions, the iTBS (*P* < 0.001) and 1-Hz (*P* < 0.001) groups exhibited significant improvements in CCAT total scores compared with their baseline scores. The results are summarized in Table 2. Regarding the CCAT subtest scores in the iTBS group, the participants’ postsession scores improved significantly in the following 8 subtests relative to the baseline scores: conversation (*P* < 0.001), description (*P* < 0.001), matching (*P* < 0.001), auditory comprehension (*P* < 0.001), expression (*P* < 0.001), reading comprehension (*P* < 0.001), imitation writing (*P* = 0.007), and spontaneous writing (*P* = 0.004) compared with baseline.

**TABLE 2:** Mean Group Data (SD) of CCAT scores obtained pre- and post-rTMS intervention.

	iTBS (*n* = 29)		1 Hz (*n* = 27)		Sham (*n* = 29)	
	Pre-rTMS	Post-rTMS	Pre-rTMS	Post-rTMS	Pre-rTMS	Post-rTMS
**CCAT score**						
Conservation	6.65 (2.73)	7.70 (2.90)***	7.26 (3.04)	7.97 (2.98)***	6.24 (3.29)	6.55 (3.41)
Description	5.78 (2.63)	6.92 (2.90)***	5.44 (2.72)	6.10 (3.08)***	5.08 (2.99)	5.38 (2.93)*
Matching	10.11 (2.19)	10.94 (1.63)***	10.75 (1.99)	11.15 (1.59)**	11.02 (2.05)	11.18 (1.99)
Auditory comprehension	7.72 (2.70)	9.11 (2.06)***	8.30 (2.37)	8.63 (2.54)	7.41 (2.83)	7.57 (2.85)
Expression	6.86 (2.50)	7.62 (2.59)***	7.05 (3.11)	7.87 (3.04)***	5.77 (3.37)	5.73 (3.41)
Reading comprehension	7.34 (2.47)	8.30 (2.49)***	7.77 (3.04)	8.54 (2.84)**	6.93 (3.07)	7.03 (3.09)
Repetition	8.10 (3.18)	8.67 (3.00)	7.83 (2.72)	8.13 (2.59)*	6.71 (3.41)	6.93 (3.37)*
Imitation writing	8.65 (3.01)	9.15 (2.70)**	9.26 (2.85)	9.94 (2.77)**	8.87 (3.20)	8.95 (3.19)
Spontaneous writing	6.15 (2.84)	6.81 (2.99)**	5.89 (2.67)	6.44 (2.85)**	5.23 (2.49)	5.41 (2.55)
Total score	67.34 (19.38)	75.22 (18.47)***	69.55 (20.93)	74.78 (20.55)***	63.26 (23.03)	64.71 (22.93)*

*Indicates significant change compared with the baseline level at P < 0.05, **P < 0.01, ***P < 0.001. (paired t-test).

*CCAT, Concise Chinese Aphasia Test; rTMS, repetitive transcranial magnetic stimulation.*

In the 1-Hz group, the participants’ postsession scores improved significantly in the following 8 subtests compared with the baseline scores: conversation (*P* < 0.001), description (*P* < 0.001), matching (*P* = 0.003), expression (*P* < 0.001), reading comprehension (*P* = 0.001), repetition (*P* = 0.029), imitation writing (*P* = 0.001), and spontaneous writing (*P* = 0.004). The participants’ scores in auditory comprehension did not improve significantly relative to the baseline scores.

We observed significant differences between the baseline and postsession description (*P* = 0.048) and repetition (*P* = 0.043) scores in the sham group.

### Intergroup Comparisons

**FIGURE 2:**
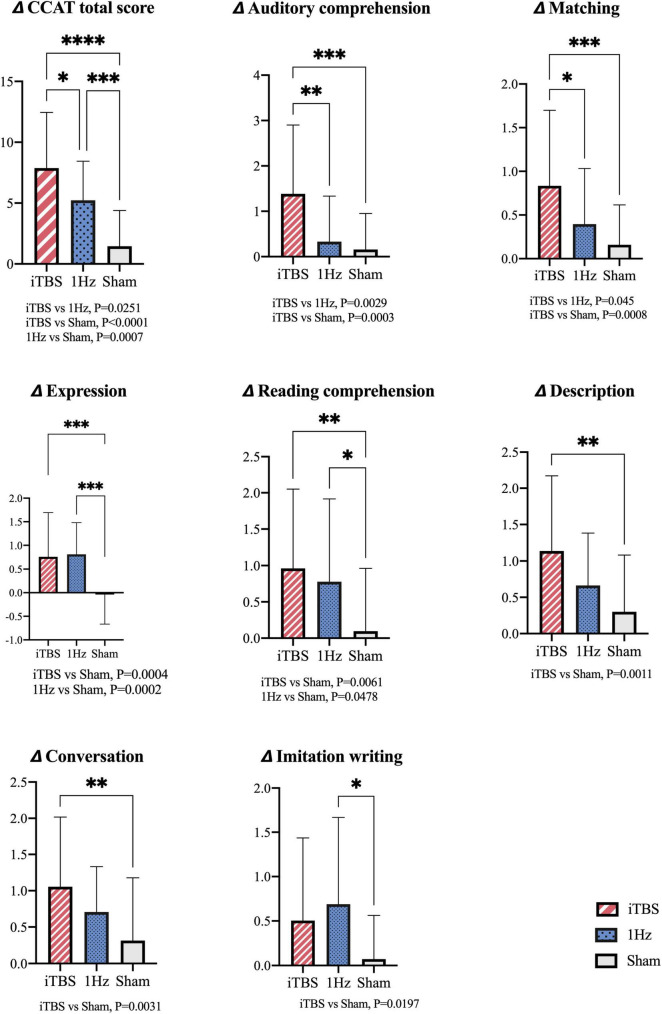
Intergroup comparisons for changes of total CCAT scores and subtest scores. *Indicates significant difference between groups at *P* < 0.05; ^**^P < 0.01; ^***^P < 0.001; ^****^P < 0.0001. ANOVA; *Post hoc* analysis: Bonferroni correction.

The ANOVA results revealed significant differences between the 3 groups in terms of total CCAT score [*F*_(2_, _82)_ = 22.58; *P* < 0.0001; Figure 2] The *post hoc* analysis results also indicated differences between the iTBS and 1-Hz groups [*F*_(2_, _82)_ = 22.58; *P* = 0.0251], iTBS and sham groups [*F*_(2_, _82)_ = 22.58; *P* < 0.0001], and 1-Hz and sham groups [*F*_(2_, _82)_ = 22.58; *P* = 0.0007].

Compared with the 1-Hz group, the iTBS group exhibited significantly superior matching [*F*_(2_, _82)_ = 7.491; *P* = 0.045] and auditory comprehension [*F*_(2_, _82)_ = 9.624; *P* = 0.0029] scores. Furthermore, compared with the sham group, the iTBS group exhibited significant improvements in conversation [*F*_(2_, _82)_ = 5.739; *P* = 0.0031], description [*F*_(2_, _82)_ = 6.926; *P* = 0.0011], and expression [*F*_(2_, _82)_ = 11.41; *P* = 0.0004] scores (under the verbal production domain) as well as in auditory comprehension [*F*_(2_, _82)_ = 9.624; *P* = 0.0003], reading comprehension [*F*_(2_, _82)_ = 5.602; *P* = 0.0061] and matching [*F*_(2_, _82)_ = 7.491; *P* = 0.0008] scores (under the perception domain).

Compared with the sham group, the 1-Hz group had superior improvements in expression [*F*_(2_, _82)_ = 11.41; *P* = 0.0002], reading comprehension [*F*_(2_, _82)_ = 5.602; *P* = 0.0478], and imitation writing [*F*_(2_, _82)_ = 4.14; *P* = 0.0197] scores.

## Discussion

In this RCT study, the ipsilesional iTBS protocol achieved a superior outcome in overall language performance compared with the LF-rTMS protocol in chronic poststroke aphasia. Both protocols demonstrated significant neuromodulation effects in the language production and perception domains, but the iTBS protocol particularly enhanced auditory semantic processing when applied in the lesioned Broca area.

### Poststroke Recovery Model and LF-rTMS

In general, sophisticated differentiated functions such as hand dexterity and language are considered to be controlled by a unilateral system in the brain (Costanzo et al., 2015). That is, these functions are dominated by a unilateral hemisphere, and a balance exists between the hemispheres in premobility status. Neuroplasticity after a brain insult is dynamic and depends on the lesion size, lesion site, lesion chronicity, remote connectivity change, and interaction with non-affected parts of the functional network (Heiss and Thiel, 2006; Cramer, 2008; Carter et al., 2010). Once the functional center is partially injured, optimal recovery relies on spared node regeneration or unmasking of potential adjacent nodes that are functionally related (Watila and Balarabe, 2015). For large lesions such as a middle cerebral artery occlusion, a previous study noted short-term contralesional hyperactivity after a stroke, and this was due to reduced ipsilesional and transcallosal inhibition (Heiss and Thiel, 2006). A study investigating poststroke aphasia recovery by using fMRI and PET assessments indicated that different right IFG subregions account for different compensatory mechanisms; for example, the right dorsal POp was recruited specifically by patients with left inferior frontal lesions, implying a possible compensatory takeover for the phonological function of the lesioned node (Turkeltaub et al., 2011). However, overactivation in the right PTr, a sequela of transcallosal disinhibition, is a maladapation and associated with incomplete recovery (Naeser et al., 2010a,^2012^; Barwood et al., 2011; Weiduschat et al., 2011). On the basis of these findings, previous studies have generally applied LF-rTMS to the right PTr region. Language improvement could be taken as evidence that right PTr overactivation could be detrimental to recovery (Turkeltaub et al., 2012; Tsai et al., 2014; Wang et al., 2014). Despite the success of LF-rTMS in enhancing naming and expression function, it did not engender significant improvements in repetition or comprehension (Angst et al., 2017). A recent meta-analysis demonstrated that the modulation target for comprehension or repetition may be located in other regions, such as the temporal lobe. The stimulus site could affect the efficacy of LF-rTMS in improving language function (Hong et al., 2021).

### Contralesional or Ipsilesional HF-rTMS

According to the hierarchical recovery model proposed by Heiss and Thiel (2006) in “severely” impaired left hemisphere networks, perilesional recruitment is inadequate for language recovery, and the right hemisphere homotopic regions appear to be capable of assuming some language functions, as indicated by their employment in ways that may mirror some aspects of language processing. However, because of genetic predisposition, developmental factors, or neuroplastic changes that lead to language lateralization, the non-dominant right hemisphere may be intrinsically less adept at language processing compared with the dominant left hemisphere counterpart. A research group recently attempted to employ HF-rTMS to promote right IFG reorganization in poststroke aphasia and reported that LF-rTMS engendered a more marked improvement than did HF-rTMS in spontaneous speech and aphasia quotients (Hu et al., 2018). This finding indicates that the upregulation of the right IFG had only a marginal effect and was even inferior to LF-rTMS, which may engender more extensive network changes, including pushing language-related activity leftward.

Therefore, targeting the left IFG with HF-rTMS may yield direct modulatory effects on language enhancement. High-frequency stimulation has previously been considered to induce seizure attacks and speech arrest during pronunciation (Malcolm et al., 2007; Oberman and Pascual-Leone, 2009; Tarapore et al., 2013). Studies investigating HF-rTMS in the motor cortex have demonstrated it to be a safe technique when applied with the appropriate stimulation frequency, intensity, duration, and intervals (Fisicaro et al., 2019). Research has reported a growing body of promising results for HF-rTMS, indicating the adequate establishment of a safety consensus for HF-rTMS. Applying excitatory HF-rTMS to the left IFG has been reported to improve language performance in healthy individuals (Mottaghy et al., 1999) and in individuals with primary progressive aphasia (Finocchiaro et al., 2006). Dammekens et al. administered 10-Hz HF-rTMS stimulation over the left IFG in a patient with non-fluent aphasia. They confirmed that after excitatory stimulation for 3 weeks, improvements in repetition, naming, and comprehension were noted, with decreased activity in the right IFG in electroencephalogram analysis (Dammekens et al., 2014).

Studies have also demonstrated the efficacy of iTBS, an ultra-high-frequency patterned rTMS protocol (Huang et al., 2005), in promoting language recovery in poststroke aphasia (Szaflarski et al., 2011, 2018). In a small study, significant linguistic gains were observed in semantic fluency performance after 2 weeks of stimulation. An association between functional improvement and stronger language lateralization to the dominant left hemisphere was shown in fMRI signals, especially in the left fronto-temporo-parietal language networks (Szaflarski et al., 2011). Griffis et al. reported improved language task–related responses (verb generation) in 8 patients after 10 sessions of iTBS treatment on the residual left IFG; although lacking a control group, general right-to-left lateralization was demonstrated by both functional and anatomical MRI data (Griffis et al., 2016). In another study, diffusion tensor imaging (DTI) of the aphasic patients also provided preliminary evidence supporting structural changes in perilesional white matter integrity near the stimulation site (Allendorfer et al., 2012). These studies have provided a rationale for the application of HF-rTMS to the lesioned IFG for treatment. Nevertheless, despite these promising results, the literature does not contain a relevant RCT with a double-blind design.

### Efficacy of Ipsilesional iTBS

In this study, we applied 10 sessions of iTBS to the left Broca area (i.e., BA45), in contrast to the LF-rTMS treatment, in which the right BA45 served as the treatment target. The outcomes of the iTBS group were superior to those of the LF-rTMS group in terms of overall performance assessed using the CCAT. Compared with the sham group, both experimental groups exhibited significant improvements in total CCAT scores and in the verbal production and perception domains. The iTBS group manifested language skill improvements in 8 subtests, whereas the LF-rTMS group exhibited such improvements in 3 subtests. In addition, a head-to-head comparison revealed that language comprehension was more susceptible to ipsilesional iTBS conditioning than to LF-rTMS.

The superior effect of the iTBS paradigm was observed in the subtests of item matching and auditory comprehension. Matching was performed by asking a participant to point to the correct picture or real object in response to an auditorily presented stimulus word. During auditory comprehension test, the participant was requested to execute commands through auditory perception. Both subtests measured semantic processing that may be associated with the activation of the left superior temporal gyrus. Our findings are consistent with those of 2 previous studies that have reported iTBS-related improvements in semantic fluency. Previous studies have reported that semantic processing was associated with increased left frontotemporal activity and decreased right IFG connectivity in pre- and post-rTMS fMRI in chronic poststroke aphasia (Szaflarski et al., 2011; Griffis et al., 2016). Although a sham-controlled group was lacking in these studies, neuroimage findings provided valuable insight into the interval neural reorganization underpinning the iTBS treatment.

The significant improvement in comprehension ability after ipsilesional PTr stimulation could be explained by the dual-stream model and redundancy recovery model (Hickok and Poeppel, 2004, 2007; Scott and Wise, 2004; Zahn et al., 2004; Specht, 2013). The dual-stream model for language processing was suggested to provide a hierarchical processing network for speech. Complementary studies combining fMRI with DTI have demonstrated that superior temporal and premotor areas were activated in the dorsal pathway through the arcuate fasciculus, which would oversee phonological and repetition processing; however, the ventral pathway, connecting the upper posterior part of the temporal lobe and the ventral IFG (PTr), is central in semantic processing and is activated during auditory comprehension (Pascual-Leone et al., 2002; Szaflarski et al., 2011; Yoon et al., 2015; Hu et al., 2018). Our iTBS target set at the PTr could closely modulate the ventral stream as well as the semantic processing network.

### Ipsilesional HF-rTMS in Extensive Left Hemispheric Lesions

Considering the massive lesions in the left frontal region in severe aphasia cases, the efficacy of language facilitation with lesioned side modulation could be challenged. Using fMRI, Zahn et al. followed global aphasic patients with extensive left middle cerebral artery infarction; they revealed a leftward asymmetry in the ventral stream for lexical and semantic processes, in contrast to the stream for acoustic, sublexical perception, which is symmetrically organized (Zahn et al., 2004). Despite the presence of extensive left-side lesions, a spared language node could be functionally activated during semantic word processing. They concluded that for comprehension recovery, the redundancy recovery noted in the damaged hemisphere, in terms of closely related functional nodes, was more essential than the takeover of function by previously unrelated areas (Zahn et al., 2004). Therefore, language comprehension may be preferentially modulated by the excitatory protocol applied on the left PTr, as demonstrated in our study. Furthermore, in addition to comprehension ability, the residual network associated with language production can be activated or reorganized through iTBS modulation, as indicated by our results.

The proposed mechanism underlying LF-rTMS modulation could be associated with the mirror neuron system in the right pars opercularis, leading to the reorganizing process in the left hemispheric networks (Kaplan et al., 2010). For the right IFG, no direct pathway was noted between the right PTr and right arcuate fasciculus. By contrast, direct pathways were present between the right pars opercularis and the right arcuate fasciculus (Kaplan et al., 2010). Suppressing the right PTr can improve the right pars opercularis through the presuming U-shaped fiber between these 2 gyri, which can in turn facilitate phonological expression by mirror neurons across bi-hemispheres (Schmahmann and Pandya, 2006; Naeser et al., 2010b). This proposed mechanism underlying the observed articulation and phonation enhancement may essentially involve less comprehension (Frey et al., 2008).

Because the neuroplastic changes following a stroke manifest as a dynamic process, time post stroke could be a consideration for the adoption of HF- or LF-rTMS. In this study, we demonstrated the stronger modulating effect exerted by HF-rTMS in chronic aphasia. In a meta-anlysis by Hong et al. (2021), LF- rTMS was more effective in subacute (≤ 3 months) patients than in chronic (> 3 months) patients (Hong et al., 2021). By contrast, preliminary evidence in a recent observational study revealed that excitatory rTMS was beneficial for chronic aphasic patients, which is in concordance with our results (Fahmy and Elshebawy, 2021). Early administration of HF-rTMS in the subacute stage can lead to the adverse side effects of overstimulation and excitotoxicity. Therefore, LF-rTMS might be preferred in the poststroke subacute stage, whereas HF-rTMS might be suitable for patients in the chronic stage. A second consideration for the use of these two paradigms is the rMT of the right motor cortex. Patients who yield lower rMT in the right motor system may benefit the most from LF-rTMS (Tsai et al., 2014). Higher rMT might indicate subcortical microangiopathy such as diabetic neurotrophic dysfunction or impaired cortical structural integrity, which were linked to an inferior modulating effect when using LF-rTMS (Tsai et al., 2014; Varkanitsa et al., 2021). In the above scenario, ipsilesional HF-rTMS might be more effective at facilitating language recovery than LF-rTMS.

## Limitations

The study has limitations. First, our sample size, despite being the largest among studies on HF-rTMS in aphasic stroke, is limited. Second, the iTBS and 1-Hz stimulation intensity levels were set to 80 and 90% of the rMT, respectively; nevertheless, some participants could not tolerate the cocontraction of facial muscles, which may have caused a painful sensation. In such circumstances, we reduced the stimulation intensity to a tolerable level. Although the lower intensity could have reduced the therapeutic effect, this occurred in both groups, which reflects practical situations. Third, we did not follow up the participants to investigate the endurance of the effects. Future studies should include a long-term follow-up to determine the substantial efficacy of ipsilesional HF-rTMS. Fourth, our outcome measurements relied on CCAT, which is an examiner-prompted assessment. Combining other kinds of patient-centered assessment tools such as the Verbal Activity Log might yield better indicators of patients’ real-world spoken language capacities (Haddad et al., 2017). Finally, because of the lack of neuroimaging and other electrophysiological evidence, further investigation is required to provide such evidence. Neuroimaging studies addressing rTMS-related microstructural and neurophysiological changes can provide insight into neuroplastic mechanisms. In the future, HF-rTMS targeting different IFG subareas other than the left PTr may hold promise in language recovery for various types of aphasia.

## Conclusion

Our study findings add to a growing body of evidence that the stimulation of the ipsilesional PTr enhances the language recovery of individuals with chronic non-fluent aphasia after a stroke. As the first RCT comparing ipsilesional iTBS, contralesional 1-Hz rTMS therapy, and a sham treatment, this study suggests that both rTMS paradigms are effective for language recovery in multiple domains. Furthermore, iTBS may be more promising than 1-Hz, especially for the comprehension aspect of patients with chronic non-fluent aphasia. Our findings may be of importance in the optimization of neuromodulation strategies.

## Data Availability Statement

The raw data supporting the conclusions of this article will be made available by the authors, without undue reservation.

## Ethics Statement

The studies involving human participants were reviewed and approved by the Institutional Review Board of Taipei Veterans General Hospital (IRB No., 201405003A). The patients/participants provided their written informed consent to participate in this study.

## Author Contributions

P-YT designed the study. T-YC, J-CW, M-YL, and P-YT acquired the data, which T-YC and P-YT analyzed. T-YC and P-YT wrote the article, which J-CW and M-YL reviewed. All authors approved the final version to be published and can certify that no other individuals not listed as authors have made substantial contributions to the manuscript.

## Conflict of Interest

The authors declare that the research was conducted in the absence of any commercial or financial relationships that could be construed as a potential conflict of interest.

## Publisher’s Note

All claims expressed in this article are solely those of the authors and do not necessarily represent those of their affiliated organizations, or those of the publisher, the editors and the reviewers. Any product that may be evaluated in this article, or claim that may be made by its manufacturer, is not guaranteed or endorsed by the publisher.
